# Atypical Presentation of Anti-Phospholipid Antibody Syndrome with Seizure and Atrial Mass

**DOI:** 10.1155/2020/8877445

**Published:** 2020-11-16

**Authors:** Rajish Sanjit Kumar Shil, Amal Abdallah Al Dhuhoori, Vipin Mughilassery Thomachan, Jamal Ali Teir, Renganathan Radhakrishnan

**Affiliations:** ^1^Medical Resident, Department of Internal Medicine, Al Ain Hospital, Al Ain, UAE; ^2^Department of Cardiology, Al Ain Hospital, Al Ain, UAE; ^3^Department of Rheumatology, Al Ain Hospital, Al Ain, UAE; ^4^Department of Neurology, Al Ain Hospital, Al Ain, UAE

## Abstract

Anti-phospholipid antibody syndrome (APS) has a broad spectrum of thrombotic and nonthrombotic clinical manifestations. The diagnosis requires a set of clinical criteria of thrombosis along with persistently positive anti-phospholipid antibody tests. In this report, we are presenting a case of APS, who is a 38-year-old male, presented with complains of seizures and found to have stroke, which on further investigation revealed to have been caused possibly from a left atrial mass. Therefore, high index of suspicion is required for the diagnosis of APS in young patients, who present with various neurological and cardiovascular manifestations, mostly secondary to thrombosis.

## 1. Introduction

Antiphospholipid syndrome is an autoimmune disorder, which may present in various ways from cutaneous manifestation, obstetric complications, and neurological and cardiac manifestation to renal involvement. There are many cardiac complications of antiphospholipid syndrome, among them are valvular dysfunction, pulmonary hypertension, myocardial infarction, intracardiac thrombi, and ventricular dysfunction [1]. These morbidities occur with the presence of anti-phospholipid antibodies (aPL), which are usually detected as cardiolipin or *β*_2_-glycoprotein I (*β*_2_-GPI) antibodies or as lupus anticoagulants [2].

Here, we describe an unusual case of APS, who presented with an episode of seizure and found to have stroke in the brain, which on further workup as part of “stroke in young” has revealed an atrial mass on echocardiography.

## 2. Case Description

A 38-year-old man, with no significant past medical illness, was brought to the emergency department initially, after he sustained an episode of generalized tonic-clonic seizure. On further questioning after being recovered from seizure, he complained of suffering from generalized headache for a duration of 10 days prior to the presentation. He denied having any weakness or numbness and denied any complains of speech or swallowing difficulties. On arrival, he was hypertensive up to 170/101 mm Hg and was hemodynamically stable otherwise. Neurological examination did not reveal any cranial nerve or cerebellar deficits and had no focal deficits, with normal symmetrical reflexes overall. Other system examinations were within normal limits.

**Figure 1:**
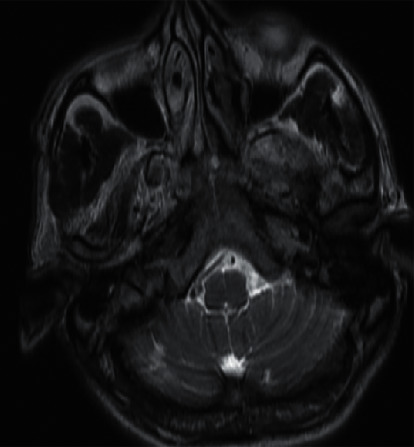
Axial view of MRI brain showing the right cerebellar infarct.

**Figure 2:**
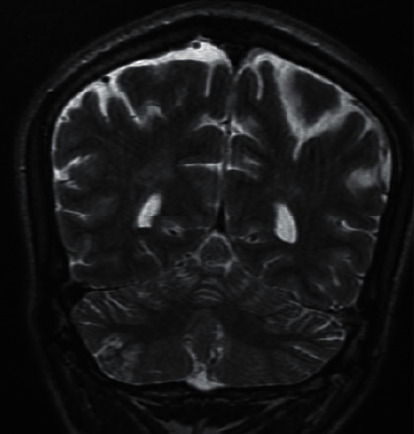
Coronal view of MRI brain showing the right cerebellar infarct.

**Figure 3:**
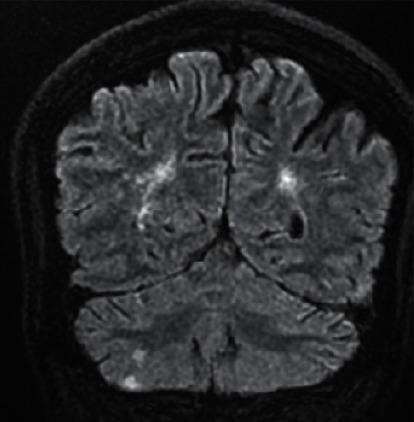
Coronal view of MRI brain showing the right cerebellar infarct.

On further investigation, initial CT brain was done, which did not reveal any abnormalities, and so he was admitted to the neurology critical care unit for further management. As a workup for seizure, MRI brain was performed, which revealed a right cerebellar small acute ischemic infarct (Figures 1–3). Subsequently, an MR angiography of the brain and neck was performed, which revealed filling defects causing focal narrowing in the origin of the internal carotid artery bilaterally. Routine brain EEG did not reveal any seizure activities.

**Figure 4:**
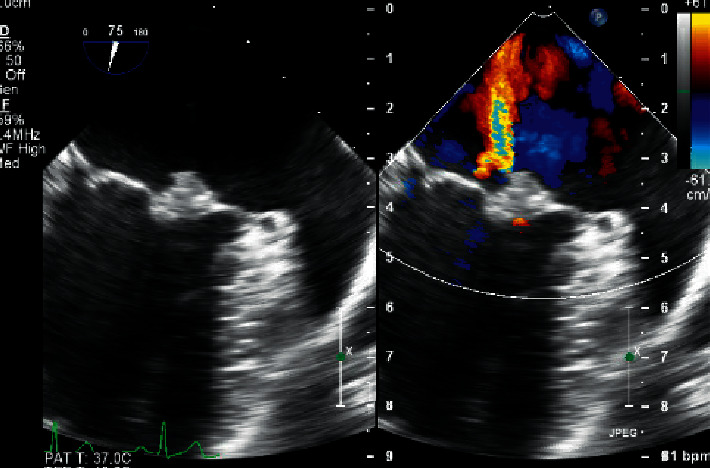
Echo view showing the left atrial mass with color Doppler.

**Figure 5:**
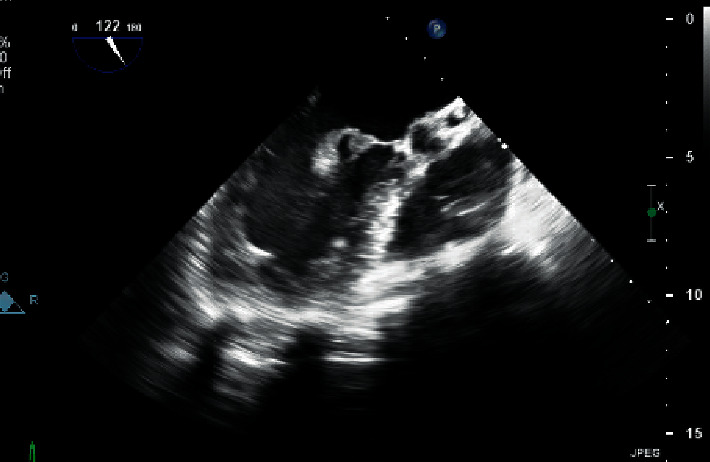
Echo view showing pedunculated left atrial mass.

Upon admission to the neurology unit, he was started on aspirin and atorvastatin for treatment of stroke. Carbamazepine was introduced in view of seizure due to focal organic cause. He was worked up thoroughly for his stroke considering his young age with complete collagen vascular disease and thrombophilia workup. He was initially found to have thrombocytopenia as low as 50,000 platelets per microliter. A transthoracic echocardiography revealed a pedunculated and lobular left atrial mass (Figures 4-5), which was measuring around 1 × 1.5 cm and was freely mobile across the mitral valve, oscillating across the left ventricular inflow. This finding was subsequently confirmed by a transesophageal echocardiography, with differential diagnosis of atrial myxoma at the top of the list. Cardiothoracic surgery opinion was sought and was planned for surgical removal of the atrial mass. Prior to transferring the patient to the surgical care center, his thrombophilia workup revealed to be positive for antibodies of antiphospholipid syndrome. Cardiolipin antibody immunoglobin G was positive in high titer >120 units/mL, and B_2_ glycoprotein IgG level was 90 units/mL. Lupus anticoagulant 1 (LA1) screen was positive for 109.30 seconds, aPTT LA was 153.8 seconds, hexagonal phase neutralizing timing was 25 seconds, LA1 mixing time was 88 seconds, and lupus anticoagulant 2 (LA2) confirmation timing was 38.46 seconds, giving a LA1/LA2 ratio of 2.84. Based on these findings, it was confirmed that dRVVT screen and aPTT are prolonged. The dRVVT mixing study was prolonged, consistent with an inhibitor effect. Hexagonal phase and dRVVT ratio were both significantly elevated, consistent with phospholipid dependence. Overall, the findings are consistent with a strong lupus anticoagulant. Lupus anticoagulants may be transient. According to the International Society of Thrombosis and Hemostasis, if positive testing is observed on two or more occasions, at least 12 weeks apart, this may be indicative of an anti-phospholipid antibody syndrome, if observed in the right clinical setting. Based on this recommendation, it was decided to repeat the testing after 12 weeks. Apart from these findings, the anti-nuclear antibody results came negative, with normal ESR and CRP levels.

**Figure 6:**
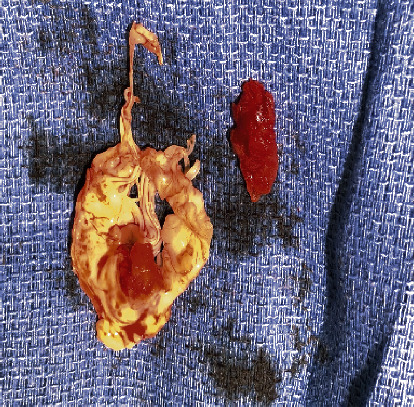
Gross specimen of the clot after the surgery.

**Figure 7:**
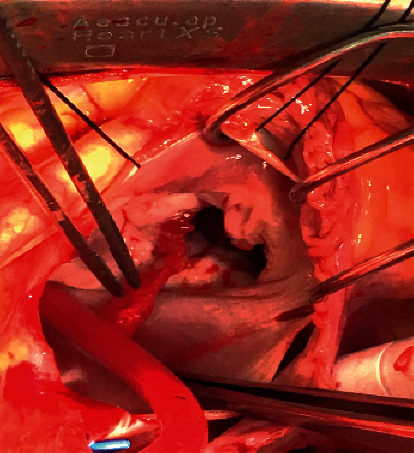
Intraoperative view, removal of the clot.

An opinion was sought from the rheumatology department, and finally the diagnosis of APS was confirmed. It was decided to start the patient on anticoagulation treatment but was delayed until the patient went for the surgery. However, on the days prior to the scheduled surgery date, he was kept on low molecular weight heparin at treatment dose and then was suspended on the operative day. The patient underwent successful surgical removal of the mass, which looked like a thrombotic clot rather than a tumor mass. Histopathological analysis confirmed that the mass is made up of a fibrinous clot, with no evidence of any tumor cells (Figures 6-7). He was started on full anticoagulation treatment with warfarin and was followed up regularly in the clinic, where he did not have any further complications from the disease. Shortly after the treatment and follow-up for 2 months, the patient repatriated back to his home country and to continue treatment there. So, it was not possible to repeat the LA and aPL studies after 12 weeks as recommended as he lost follow-up with us.

## 3. Discussion

Antiphospholipid syndrome is defined according to the clinical and laboratory criteria established by the updated international consensus (Sydney) classification (ICS) for definitive diagnosis, which requires a history of vascular thrombosis and/or pregnancy morbidity, associated with the persistent presence of lupus anticoagulants and/or anti-cardiolipin antibodies of the immunoglobulin G and/or immunoglobulin M isotype, positive in medium or high titers (i.e., >40 GPL or MPL or >99^th^ percentile), and/or anti-*β*_2_-glycoprotein-1 (a-*β*_2_-GPI) (IgG and/or IgM) > 99^th^ percentile in the blood. These aPL should be persistent, defined as being present on two or more consecutive occasions at least 12 weeks apart [2]. APS usually is classified as primary and secondary, respectively, according to the absence or presence of an underlying disease as well as other autoimmune, malignancy, or drug-induced disorders. In both instances, every organ can be involved by thrombotic events. In a recent study of a cohort of 1000 patients affected by primary and secondary APS, cardiac involvement was observed in 27% of cases [3], mostly characterized by valvular and coronary artery disease. The most frequent echocardiographic findings are either valve vegetations or leaflet thickening. The literature reports only a few cases of intracardiac thrombosis [4] associated with primary APS, observed in all cardiac chambers, provoking either pulmonary or systemic embolism.

The major target of anti-phospholipid antibodies is *β*_2_-glycoprotein I (*β*_2_-GPI) in APS. Stroke and transient ischemic attack are the most common arterial events in patients with APS. The major nonthrombotic manifestations of anti-phospholipid antibody positivity include valvular heart disease, livedo reticularis, anti-phospholipid antibody-related nephropathy, thrombocytopenia, hemolytic anemia, and cognitive dysfunction.

Our patient was diagnosed to have APS based on the features of high positive anti-cardiolipin antibody IgG and B2 glycoprotein IgG, stroke, thrombocytopenia, and abnormal echocardiography findings. So far, only few cases of APS-associated right atrial thrombosis have been reported [5–7]. In our patient, it was difficult to distinguish between the left atrial myxoma as the primary tumor of the heart and cardiac thrombosis based on preoperative echocardiographic and angiographic findings. It was decided to go for surgical resection of the cardiac mass over any further imaging investigations, as it would help reaching the definitive diagnosis of the cardiac mass based on histopathology faster than investigating through other imaging modalities. This decision was considered clearly based on the severely affected mitral valve status, the size and mobility of the intracardiac mass, and also based on the suspicion of embolic stroke in the patient although the stroke could be due to the primary APS itself as the mass turned out to be a thrombotic clot rather than an atrial myxoma. Other cardiac manifestations of the APS include aseptic valve lesions, particularly of the tricuspid and aortic valves, coronary artery disease, and ventricular dysfunction because of the myocardial microthrombosis [2].

In our patient, long-term anticoagulation was started with vitamin K antagonist (VKA) as it is a standard treatment for the prevention of recurrent thrombosis in definitive APS [8]. Taking this into context, using VKA for some patients with APS could be difficult as the INR values may fluctuate based on the strong LA, depending on the prothrombin time reagent being used for the test. However, after fully explaining the risks and benefits to the patient, it was decided to start VKA treatment as per the patient and physician's preference. It is true that there is no requirement for monitoring the anticoagulation effects routinely for the direct oral anticoagulants (DOACs), like factor Xa inhibitors (rivaroxaban/apixaban) or the thrombin inhibitor (dabigatran), but, however, the recent case reports on patients with APS experiencing recurrent thromboembolic events while on therapy with DOACs have questioned their usefulness as safe and efficacious alternatives in this highly prothrombotic disorder [9, 10]. Further studies would be required to assess the eligibility and safety of using DOACs in patients with definitive antiphospholipid syndrome.

## 4. Conclusion

APS can present with neurological and cardiac manifestations, and therefore high index of suspicion is necessary for diagnosis of the disease in young patients with thrombosis as it can affect both short- and long-term treatment plan and prognosis. Therefore, in patients presenting with neurological symptoms like seizures, weakness, and radiological diagnosis of stroke in a young patient, where atrial masses could be thought to be the cause of stroke, they should be screened for any concomitant findings of thrombocytopenia and/or aPTT prolongation which should raise the suspicion of thrombotic vasculopathy, specifically APS, to be the primary cause of the clinical presentation.

## Data Availability

The radiographical imaging and laboratory data were collected from electronic medical records and were used to support the findings of this study for publication of this individual case report.

## Abbreviations

APS: Anti-phospholipid antibody syndrome
aPL: Anti-phospholipid antibodies
*β*_2_-GPI: *β*_2_-Glycoprotein I
CT: Computed tomography
MRI: Magnetic resonance imaging
EEG: Electroencephalogram
ESR: Erythrocyte sedimentation rate
CRP: C-reactive protein
aPTT: Activated partial thromboplastin time
VKA: Vitamin K antagonist
DOAC: Direct oral anticoagulant
LA: Lupus anticoagulant
dRVVT: Dilute Russell's viper venom test.
